# Functional assessment using 3D movement analysis can better predict health-related quality of life outcomes in patients with adult spinal deformity: a machine learning approach

**DOI:** 10.3389/fsurg.2023.1166734

**Published:** 2023-05-03

**Authors:** Elio Mekhael, Rami El Rachkidi, Renee Maria Saliby, Nabil Nassim, Karl Semaan, Abir Massaad, Mohamad Karam, Maria Saade, Elma Ayoub, Ali Rteil, Elena Jaber, Celine Chaaya, Julien Abi Nahed, Ismat Ghanem, Ayman Assi

**Affiliations:** ^1^ Faculty of Medicine, Saint Joseph University of Beirut, Beirut, Lebanon; ^2^ Technology Innovation Unit, Hamad Medical Corporation, Doha, Qatar; ^3^ Institut de Biomécanique Humaine Georges Charpak, Arts et Métiers ParisTech, Angers, France

**Keywords:** adult spinal deformity, machine learning, 3D movement analysis, gait, follow-up, functional assessment, health-related quality of life

## Abstract

**Introduction:**

Adult spinal deformity (ASD) is classically evaluated by health-related quality of life (HRQoL) questionnaires and static radiographic spino-pelvic and global alignment parameters. Recently, 3D movement analysis (3DMA) was used for functional assessment of ASD to objectively quantify patient's independence during daily life activities. The aim of this study was to determine the role of both static and functional assessments in the prediction of HRQoL outcomes using machine learning methods.

**Methods:**

ASD patients and controls underwent full-body biplanar low-dose x-rays with 3D reconstruction of skeletal segment as well as 3DMA of gait and filled HRQoL questionnaires: SF-36 physical and mental components (PCS&MCS), Oswestry Disability Index (ODI), Beck's Depression Inventory (BDI), and visual analog scale (VAS) for pain. A random forest machine learning (ML) model was used to predict HRQoL outcomes based on three simulations: (1) radiographic, (2) kinematic, (3) both radiographic and kinematic parameters. Accuracy of prediction and RMSE of the model were evaluated using 10-fold cross validation in each simulation and compared between simulations. The model was also used to investigate the possibility of predicting HRQoL outcomes in ASD after treatment.

**Results:**

In total, 173 primary ASD and 57 controls were enrolled; 30 ASD were followed-up after surgical or medical treatment. The first ML simulation had a median accuracy of 83.4%. The second simulation had a median accuracy of 84.7%. The third simulation had a median accuracy of 87%. Simulations 2 and 3 had comparable accuracies of prediction for all HRQoL outcomes and higher predictions compared to Simulation 1 (i.e., accuracy for PCS = 85 ± 5 vs. 88.4 ± 4 and 89.7% ± 4%, for MCS = 83.7 ± 8.3 vs. 86.3 ± 5.6 and 87.7% ± 6.8% for simulations 1, 2 and 3 resp., *p* < 0.05). Similar results were reported when the 3 simulations were tested on ASD after treatment.

**Discussion:**

This study showed that kinematic parameters can better predict HRQoL outcomes than stand-alone classical radiographic parameters, not only for physical but also for mental scores. Moreover, 3DMA was shown to be a good predictive of HRQoL outcomes for ASD follow-up after medical or surgical treatment. Thus, the assessment of ASD patients should no longer rely on radiographs alone but on movement analysis as well.

## Introduction

A global aging of the worldwide population is taking place as the proportion of the population over 60 years old is predicted to increase to 22% in 2,050 and 32% in 2,100 (1). This is known to be associated with an increase in multiple degenerative disorders, especially in the musculoskeletal system, such as adult spinal deformity (ASD), an already highly prevalent pathology affecting between 32% and 68% of subjects older than 65 years (2). Patient care and research on spinal deformity has gained a lot of attention during the last decade because of the economic impact of the pathology on the health system (3) and its burden on the psychosocial state of the patient (4, 5).

Adult spinal deformity (ASD) consists of a variety of postural and spino-pelvic alterations of the lumbar or thoracolumbar spine, involving one or more of the three planes (6). Until now, treatment planning for patients with spinal deformity is based on clinical examination and static radiographic assessment of the deformity (7, 8), a trivial evaluation method, knowing that a spinal deformity is an orthopedic pathology that requires a standard radiography for its assessment.

Since any orthopedic or surgical intervention is mainly driven by the improvement of patient's quality of life, HRQoL scores, both in their physical and mental components, became key indicators to evaluate patients' follow-up (4). The physical components specifically are related to the ability of patients to live normally and exhibit an adequate level of independence during daily life activities such as walking, sitting and standing, climbing stairs, etc. Therefore, this functional component is necessary in order to efficiently understand the overall patient's quality of life.

More recently, many authors discussed the necessity of functional evaluation (9–11). Recent studies based on 3D movement analysis evaluation have shown the importance of dynamic assessment in ASD during walking or other daily life activities (10–13). While some of these studies have shown a relationship between HRQoL scores and kinematic parameters (10–13), the role of functional assessment in illustrating the quality of life of the patient is still unknown. Thus, the aim of this study was to determine the role of both static and functional assessments in the prediction of HRQoL outcomes using machine learning methods.

## Methods

### Data collection and acquisitions

This is an IRB approved (CEHDF1259) single-center prospective study. Inclusion criteria for the subjects with ASD were age over 20 years, self-reported back pain, and any of the following radiographic criteria: frontal Cobb >20°, sagittal vertical axis (SVA) >5 cm, pelvic tilt (PT) >25°, pelvic incidence – lumbar lordosis mismatch (PI-LL) >10° and/or thoracic kyphosis (TK) >60°. Exclusion criteria included any neurological disease or lower limb pathology that might alter patient's movement. A group of ASD was followed up after orthopedic, medical or surgical treatment.

A group of asymptomatic control subjects with no history of pain or surgery at the levels of the lower limbs or the spine, no musculoskeletal disorders, and no history of degenerative joint diseases was included. All subjects signed a written informed consent form.

**Figure 1:**
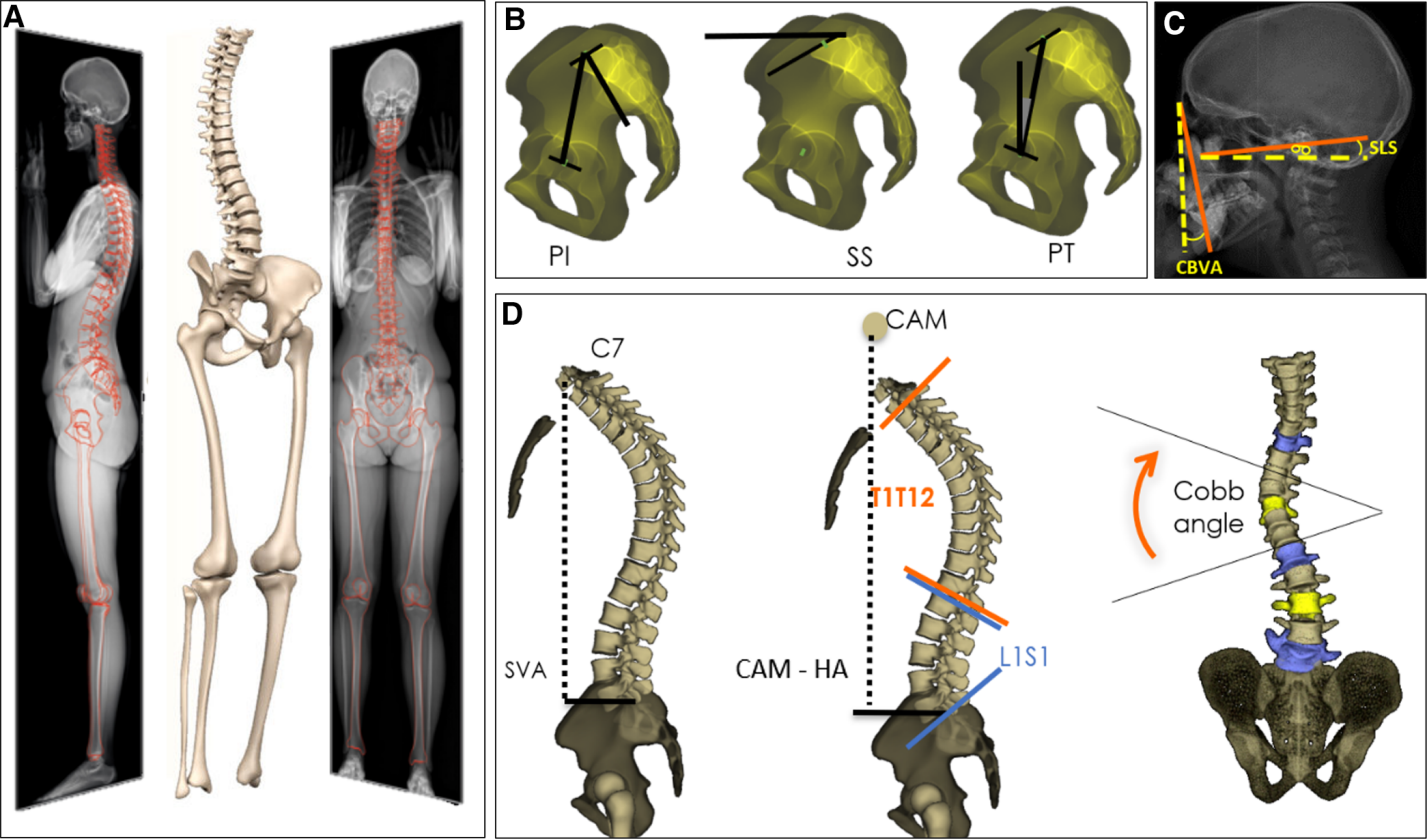
3D reconstructions of the spine based on biplanar X-rays (**A**), with calculation of spino-pelvic and global alignment parameters (**B**, **C**, **D**).

Demographic data was collected for both ASD and controls (age, height, weight and sex). Subjects underwent full-body low-dose biplanar x-rays (EOS® Imaging, Paris, France) in the free-standing position (14, 15). Three-dimensional spine reconstruction were performed by trained operators using a dedicated software (SterEOS®, EOS® Imaging, Paris, France; Figure 1A), from which the following 3D spino-pelvic and global postural parameters were generated: pelvic incidence (PI), sacral slope (SS), pelvic tilt (PT), L1S1 lordosis (LL), PI-LL mismatch, T1T12 kyphosis (TK), sagittal vertical axis (SVA), CAM-HA (the horizontal offset from the plumbline dropped from the center of the acoustic meati to the hip axis) (16), as well as knee flexion/extension and pelvic shift. The coronal Cobb angle and the apical vertebral rotation (AVR) were also calculated. Additionally, the following cervical and horizontal gaze parameters were measured: chin brow vertical angle (CBVA), the slope of line of sight (SLS), the cervical SVA distance, C0C2 and C2C7 angles (Figures 1B,D) (17).

**Figure 2:**
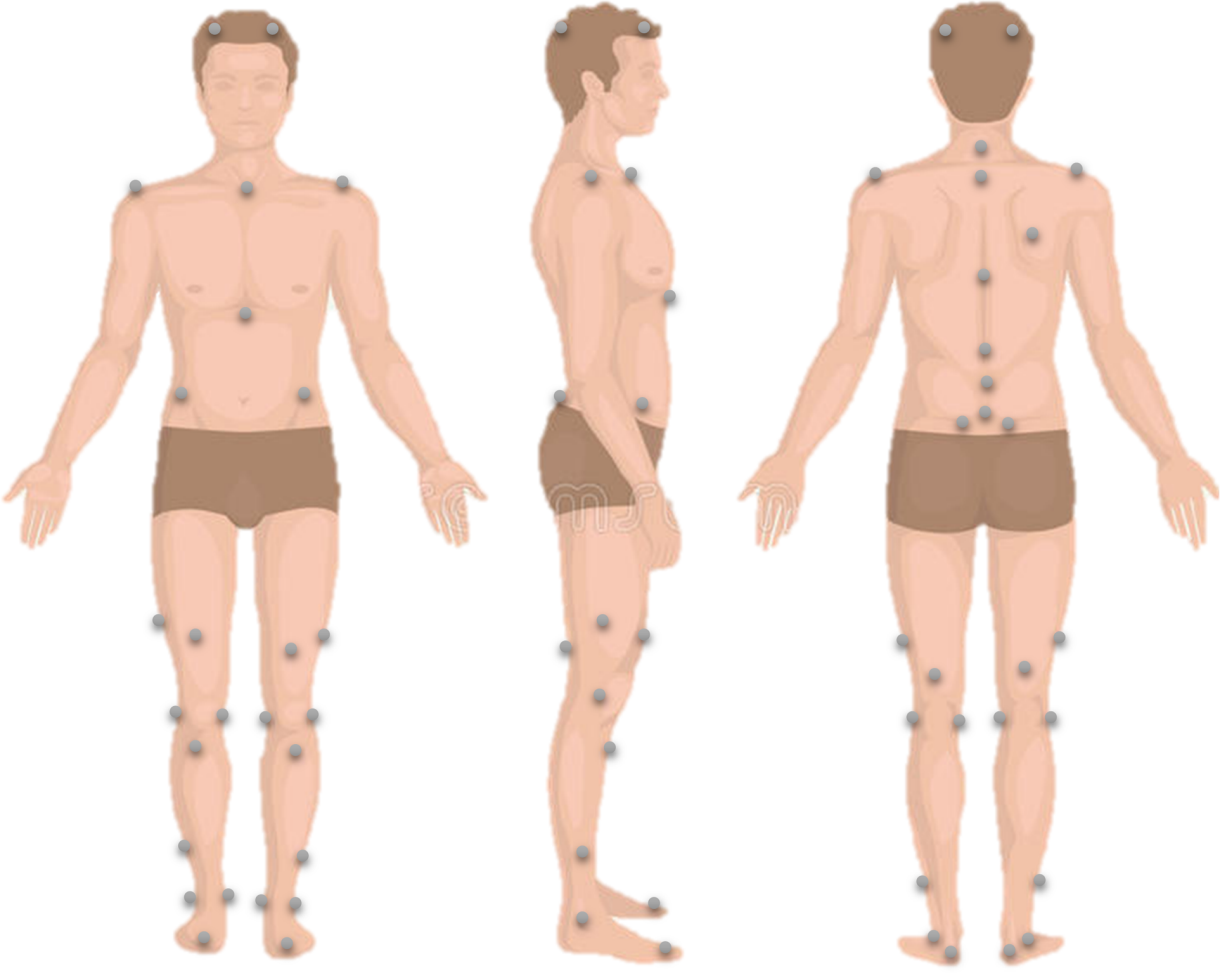
Reflective markers’ placement for lower limb and trunk kinematics calculation during walking.

All subjects underwent gait analysis using 8 optoelectronic cameras (Vicon®, Oxford, UK). In total, 41 reflecting markers were placed over bony landmarks based on the Davis and Leardini protocols for lower limb and trunk kinematics respectively (Figure 2) (18, 19). All subjects were asked to walk barefoot at a self-selected speed on a 10-m walkway several times. Classic kinematic parameters were calculated using Nexus® and Procalc® (Vicon®, Oxford, UK). The collected parameters consisted of time-distance parameters and kinematic waveforms during the gait cycle (with the calculation of the mean, maximum, minimum and range of motion ROM) of the trunk, spine, pelvis and lower limb joints in the three planes. The Gait Deviation Index (GDI), which is a subject specific score evaluating the overall pelvis and lower limb kinematic alterations during walking (varies between 0 and 100; 100 represents normal gait and deteriorates with decreasing scores) (20), was also calculated.

**Figure 3:**
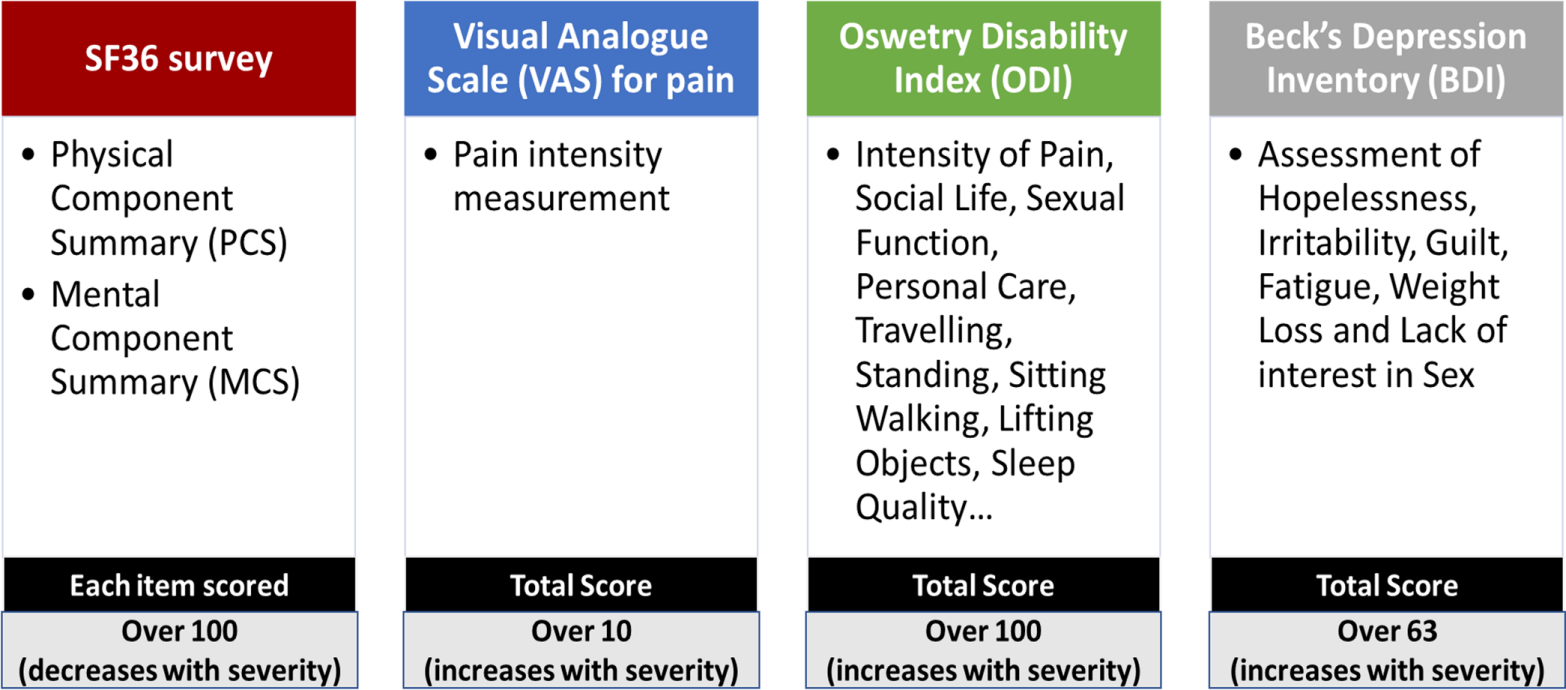
Health-related quality of life questionnaires including SF-36 survey, visual analogue scale for pain, oswetry disability index and Beck's depression inventory.

All subjects filled the following HRQoL questionnaires: visual analog scale (VAS) for pain, the Oswestry Disability Index (ODI), the Short Form Health Survey (SF-36) evaluating both physical (PCS) and mental (MCS) health components, and Beck's Depression Inventory (BDI) (Figure 3).

### Statistical analysis

In order to investigate differences in demographics, radiographic, kinematic parameters and HRQoL outcomes between ASD and controls, a Mann–Whitney's *U* test or Student's *t*-test was applied, depending on data normality (assessed using Shapiro–Wilk's test).

**Figure 4:**
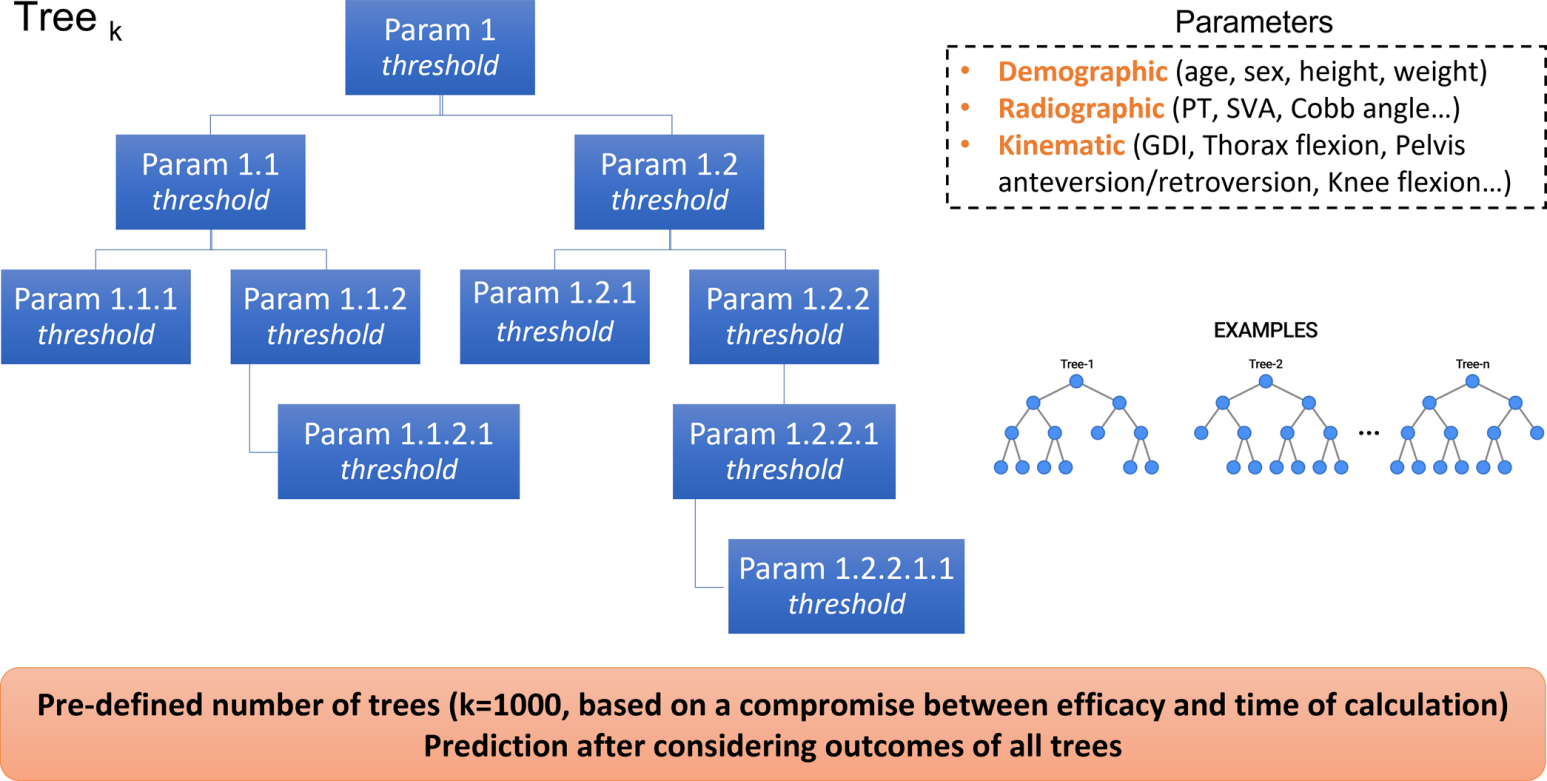
Visualization of a random forest machine learning model.

In order to predict HRQoL outcomes based on radiographic and/or kinematic parameters, a machine learning model based on random forest regression was used. A random forest is an estimator that fits an operator-defined number of classifying decision trees on various sub-samples of the training dataset. Prediction is made by evaluating the information of the ensemble of the decision trees, to improve the accuracy of prediction and control over-fitting (Figure 4).

**Figure 5:**
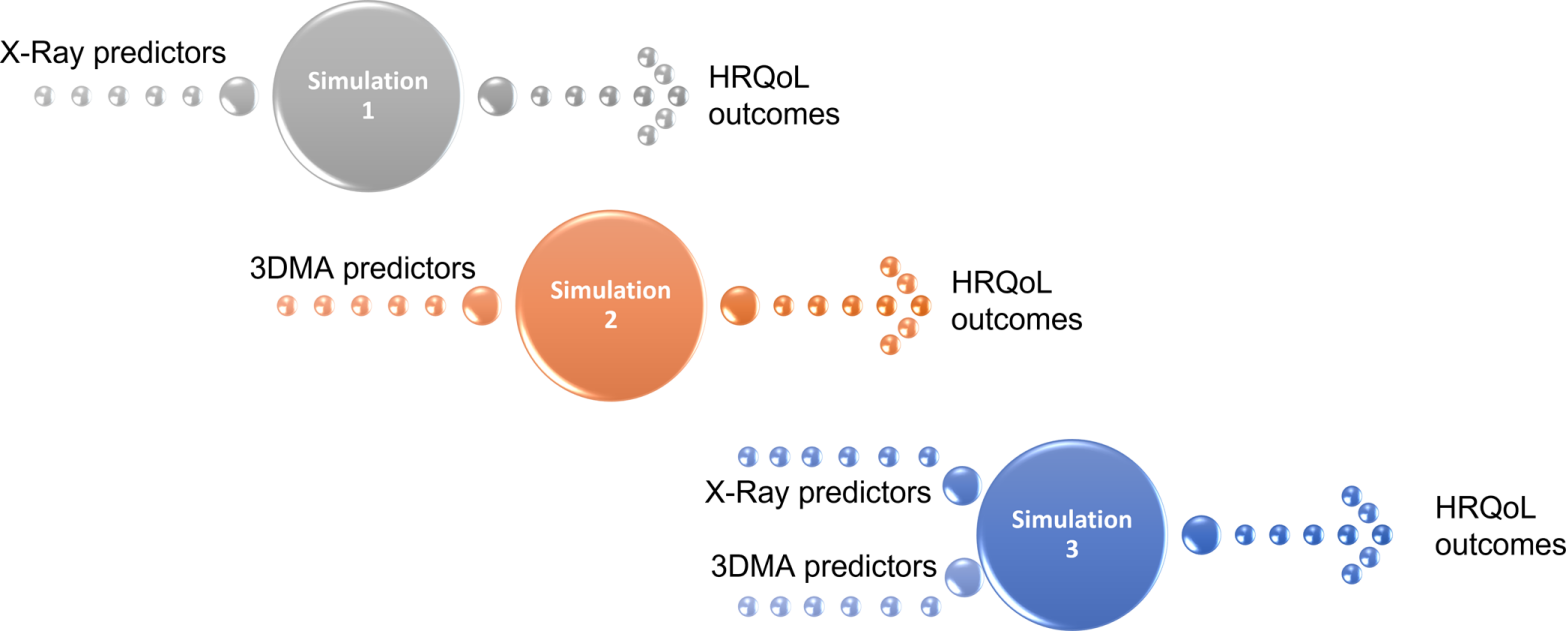
The 3 simulations performed to predict HRQoL outcomes with the following inputs: x-ray predictors (simulation 1), 3D movement analysis predictors (simulation 2) and x-ray + 3D movement analysis predictors (simulation 3).

Three simulations were conducted with different inputs that the model crosses with every single tree in the forest, and then averages the response over all the trees in order to predict one common output: HRQoL outcomes (Figure 5). The number of trees selected was 1,000 trees, a choice made with the main aim to obtain maximal accuracy of prediction, with the less time of calculation possible.

The first simulation predicted HRQoL outcomes based on the radiographic spino-pelvic parameters. The second simulation predicted HRQoL outcomes based on the kinematic parameters. Whereas the third simulation predicted HRQoL outcomes based on both radiographic spino-pelvic and kinematic parameters.

**Figure 6:**
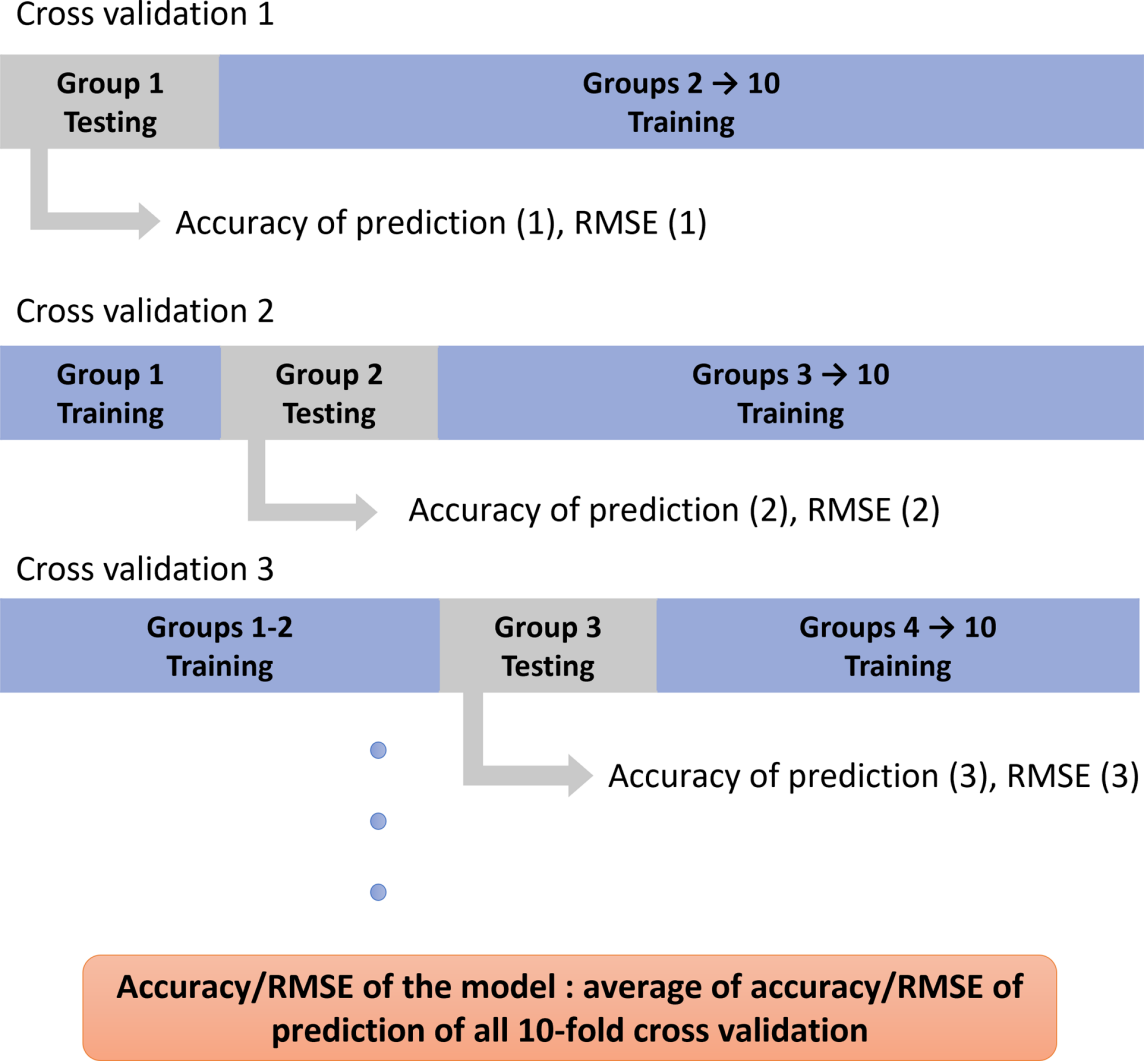
Visualization of the 10-fold cross validation of the model.

The accuracy of prediction defined as the percentage of correct predictions of each HRQoL outcome was calculated as follow: if the predicted value lies within the range of the expected value ± the margin of error [3 points for all HRQoL outcomes except 1 point for VAS for pain (21)], then it was considered as an accurate prediction, otherwise as inaccurate. The percentage of accurate predictions among all predicted values was reported. The root mean squared error (RMSE) was also calculated to quantify the difference between predicted and expected values. Subjects are divided randomly into 10 groups and a ten-fold cross validation is applied to ensure that every group of patients is used once for testing and 9 times for training. Therefore, 10 accuracy and 10 RMSE values are obtained for each HRQoL outcome. The average of all 10 values represents the average accuracy and RMSE of the model for each outcome (Figure 6).

The 10 accuracy and the 10 RMSE values obtained for each parameter were then compared between simulations using a one-way ANOVA test or Kruskal–Wallis test depending on the normality of data.

In order to investigate the possibility of predicting HRQoL outcomes after medical, orthopedic and surgical treatment, the 3 aforementioned trained models were performed on the group of ASD after follow-up.

Level of significance was set to 0.05 and was adjusted if necessary using a Bonferroni correction method. Statistical analysis was conducted using SPSS® (IBM®, New York, USA; version 2017).

## Results

### Population

Data was collected from 173 ASD patients (52 ± 19 years, 123 F) and 57 control subjects (50 ± 9 years, 35 F) with similar age distribution (*p* > 0.05, Table 1). Out of the 173 ASD patients, 63 had frontal deformity (frontal Cobb >20°), 66 had hyperkyphosis (thoracic kyphosis >60°), and 44 were enrolled based on their sagittal malalignment (SVA > 5 cm, PI-LL > 10° and/or PT > 25°). In total, 30 patients were followed up 6 months to two years after medical treatment (*N* = 7), orthopedic treatment (physical therapy: *N* = 2) and surgery (*N* = 21).

**Table 1:** Demographic comparison between groups.

Demographics	ASD (*n* = 173)	Controls (*n* = 57)	*p*-value
Age (years)	52 ± 19	50 ± 9	0.05
Weight (Kg)	74 ± 16	75 ± 13	0.47
Height (cm)	163 ± 10	167 ± 9	**0**.**001**
Sex			
Male	53 (29%)	22 (39%)	**<0**.**001**
Female	123 (71%)	35 (61%)	

Bold: significant *p*-value (*p* < 0.05).

Comparison of radiographic, kinematic parameters and HRQoL outcomes are displayed in Tables 2, 3, 4 respectively.

**Table 2:** Radiographic parameters comparison between groups.

Radiographic parameters	ASD (*n* = 173)		Controls (*n* = 57)		*p*-value
	Mean	SD	Mean	SD	
SVA (mm)	28.3	56.7	−8.0	22.2	**<0** **.** **001**
CAM plumb line (mm)	6.0	58.4	−25.2	32.4	**<0**.**001**
Spino Sacral Angle (°)	121.9	16.9	130.5	8.4	**<0**.**001**
T9 Tilt (°)	13.2	5.6	13.2	4.1	0.92
PI (°)	53.1	12.3	49.2	11.4	**0**.**009**
SS (°)	34.2	11.5	37.1	9.4	0.09
PT (°)	18.9	10.9	12.3	6.7	**<0**.**001**
L1S1 (°)	53.0	20.4	61.3	10.9	**0**.**01**
PI-LL (°)	0.1	20.2	−12.1	10.2	**<0**.**001**
T4T12 (°)	46.4	18.9	44.0	13.4	0.5
T1T12 (°)	50.9	17.9	49.6	14.6	0.88
Cobb angle (°)	17.4	17.4	6.1	9.6	**<0**.**001**
Apical vertebral rotation (°)	7.6	8.9	2.2	5.4	**<0**.**001**
Knee flexion (°)	1.8	9.5	−3.2	5.1	**<0**.**001**
CBVA (°)	3.1	5.5	1.9	6.4	0.21
SLS (°)	4.7	7.6	5.7	6.7	0.47
CO-C2 (°)	30.0	12.2	34.0	8.9	**0**.**02**
C2-C7 (°)	10.2	16.0	5.2	13.4	**0**.**03**
cSVA (°)	4.7	7.1	10.5	10.1	**<0**.**001**

Bold: significant *p*-value (*p* < 0.05).

**Table 3:** Kinematic parameters comparison between groups.

Kinematic parameters	ASD (*n* = 173)		Controls (*n* = 57)		*p*-value
	Mean	SD	Mean	SD	
Gait Deviation Index (GDI)	87.5	14.6	94.5	12.1	**0** **.** **001**
Mean Pelvic Tilt (°)	9.7	7.8	11.7	7.3	0.17
ROM Pelvic Tilt (°)	3.8	1.6	3.6	1.2	0.64
Mean Pelvic Obliquity (°)	0.0	2.4	0.1	1.3	0.862
ROM Pelvic Obliquity (°)	8.0	4.0	9.7	3.4	**0**.**001**
Mean Pelvic Rotation (°)	0.4	3.7	1.3	2.6	0.05
ROM Pelvic Rotation (°)	10.5	4.6	11.3	3.8	0.06
Mean hip Flexion/Extension (°)	15.7	8.8	16.9	8.5	0.438
ROM hip Flexion/Extension (°)	41.2	7.3	44.7	5.2	**0**.**002**
Mean hip Internal/External Rotation (°)	−3.9	13.2	−0.7	9.5	0.10
ROM hip Internal/External Rotation (°)	33.0	14.6	33.8	12.6	0.47
Mean hip Abduction/Adduction (°)	−0.5	4.4	−0.5	3.8	0.99
ROM hip Abduction/Adduction (°)	13.7	4.2	14.4	3.6	0.15
Mean Knee Flexion/Extension (°)	21.8	5.8	21.2	5.4	0.495
ROM Knee Flexion/Extension (°)	55.6	8.7	59.8	7.9	**0**.**001**
Mean Dorsal/Plantar Flexion (°)	6.7	4.8	5.5	5.0	0.13
ROM Dorsal/Plantar Flexion (°)	30.5	8.5	29.1	6.4	0.50
Mean Foot External/Internal Rotation (°)	−11.8	9.2	−10.3	5.9	0.19
ROM Foot External/Internal Rotation (°)	10.4	4.6	10.0	4.1	0.60
Mean Thorax Flexion/Extension (°)	7.8	11.2	4.6	4.7	0.356
ROM Thorax Flexion/Extension (°)	3.1	1.3	3.2	1.2	0.847
Mean Shoulder-Pelvis Rotation (°)	1.3	3.7	0.9	3.2	0.635
ROM Shoulder-Pelvis Rotation (°)	14.0	5.6	16.1	4.4	**0**.**002**
Walking Speed (m/s)	1.0	0.3	1.2	0.2	**<0**.**001**
Cadence (step/min)	102.7	14.2	111.9	12.3	**<0**.**001**
Step Length (m)	0.5	0.1	0.6	0.1	**<0**.**001**

Bold: significant *p*-value (*p* < 0.05).

**Table 4:** Health related quality of life (HRQoL) outcomes comparison between groups.

HRQoL outcomes	ASD (*n* = 173)		Controls (*n* = 57)		*p*-value
	Mean	SD	Mean	SD	
PCS (SF36)	40.9	9.6	49.0	8.4	**<0** **.** **001**
MCS (SF36)	51.6	8.8	53.6	7.6	0.27
Physical Functioning PF	42.1	11.9	49.4	11.9	**<0**.**001**
Role Physical RP	38.7	6.4	46.5	9.1	**<0**.**001**
Bodily Pain BP	45.1	9.6	53.3	7.8	**<0**.**001**
General Health GH	47.0	10.2	52.0	8.9	**0**.**001**
Vitality VT	47.1	11.6	51.9	11.1	**0**.**01**
Social Functioning SF	49.9	10.3	53.3	8.2	0.05
Role Emotional RE	42.2	5.7	48.8	8.1	**<0**.**001**
Mental Health MH	54.1	11.3	55.3	10.0	0.86
VAS for pain	5.5	2.7	4.3	2.4	**0**.**002**
ODI	28.8	18.4	19.1	12.6	**<0**.**001**
BDI	10.2	7.7	7.3	6.2	**0**.**005**

Bold: significant *p*-value (*p* < 0.05).

### Machine learning model results

In the first simulation, when spino-pelvic parameters were given alone as inputs, a median accuracy of HRQoL outcome prediction of 83.4% was recorded, with a maximum of 97% (for Role Emotional component in SF36) and a minimum of 68% (for ODI). A median RMSE of HRQoL outcome prediction of 1.68 was recorded, with a maximum of 2.68 (for ODI) and a minimum of 0.45 (for VAS for pain).

In the second simulation, when kinematic gait parameters were given alone as inputs, a median accuracy of HRQoL outcome prediction of 84.7% was recorded, with a maximum of 99% (for Role Emotional component in SF36) and a minimum of 70.8% (for ODI). A median RMSE of HRQoL outcome prediction of 1.55 was recorded, with a maximum of 2.53 (for ODI) and a minimum of 0.38 (for VAS for pain).

In the third simulation, when both kinematic and spinopelvic parameters were given as inputs, a median accuracy of HRQoL outcome prediction of 87% was recorded, with a maximum of 98.3% (for Role Physical component in SF36) and a minimum of 71.7% (for ODI). A median RMSE of HRQoL outcome prediction of 1.56 was recorded, with a maximum of 2.54 (for ODI) and a minimum of 0.38 (for VAS for pain).

In terms of accuracy of prediction, simulations 2 and 3 had comparable results for all HRQoL outcomes (Table 5). Simulations 2 and 3 showed statistically significant higher predictions of HRQoL outcomes when compared to Simulation 1. For instance, PCS was predicted at 88% and 89% of accuracy in simulations 2 and 3 but at 85% in simulation 1 (*p* < 0.005). ODI was predicted at 71% of accuracy in simulations 2 and 3 but at 68% in simulation 1 (*p* < 0.05).

**Table 5:** Accuracy of prediction of HRQoL outcomes for primary ASD and controls over the 10-fold cross validation, with comparison between the 3 simulations.

HRQoL outcomes	Simulation 1 (x-ray)				Simulation 2 (3D movement analysis)				Simulation 3 (x-ray + 3D movement analysis)				Between-simulation comparisons			
	Average	SD	95% C.I.		Average	SD	95% C.I.		Average	SD	95% C.I.		*p*-value	Simulation 1 vs. Simulation 2	Simulation 1 vs. Simulation 3	Simulation 2 vs. Simulation 3
PCS (SF36)	85.0	5.0	81.9	88.1	88.4	4.0	85.9	90.8	89.7	3.9	87.3	92.1	**<0.001**	*	*	
MCS (SF36)	83.7	8.3	78.5	88.8	86.3	5.6	82.9	89.8	87.7	6.8	83.5	91.9	**0**.**001**	*	*	
Physical Functioning PF	77.7	8.6	72.4	83.1	80.4	3.7	78.1	82.6	81.7	5.0	78.6	84.8	**<0**.**001**	*	*	
Role Physical RP	92.4	5.6	88.9	95.9	98.0	1.4	97.1	98.9	98.3	1.5	97.4	99.2	**<0**.**001**	*	*	
Bodily Pain BP	83.4	5.7	79.9	86.9	85.4	5.2	82.1	88.6	87.7	3.3	85.6	89.8	**<0**.**001**	*	*	
General Health GH	83.0	6.0	79.3	86.8	84.0	4.8	81.1	87.0	86.4	4.1	83.8	88.9	**<0**.**001**	*	*	
Vitality VT	74.4	6.9	70.1	78.6	76.4	12.3	68.7	84.0	77.7	6.0	74.0	81.4	**0**.**01**		*	
Social Functioning SF	83.4	6.6	79.3	87.4	84.0	6.0	80.3	87.8	85.0	6.3	81.1	88.9	**0**.**004**		*	
Role Emotional RE	97.0	1.4	96.1	97.9	99.0	1.5	98.1	99.9	98.0	1.4	97.1	98.9	**<0**.**001**	*	*	
Mental Health MH	76.7	10.3	70.3	83.1	76.7	7.8	71.9	81.6	77.1	10.3	70.7	83.4	0.07			
VAS for pain	72.7	9.7	66.7	78.7	74.1	8.9	68.6	79.6	74.7	8.5	69.4	80.0	**0**.**002**	*	*	
ODI	68.0	7.1	63.6	72.4	70.8	9.5	64.9	76.7	71.7	7.0	67.4	76.1	**0**.**005**	*	*	
BDI	89.7	3.8	87.3	92.0	91.0	3.2	89.0	93.0	91.3	2.4	89.8	92.9	**<0**.**001**	*	*	

Bold or *p < 0.05.

In terms of RMSE of prediction, simulations 2 and 3 had comparable results for all HRQoL outcomes (Table 6). Simulations 2 and 3 showed statistically significant higher predictions of HRQoL outcomes when compared to Simulation 1. For instance, RMSE for PCS component in SF36 was 1.53 and 1.54 in simulations 2 and 3 but 1.68 in simulation 1 (*p* < 0.05). RMSE for ODI was 2.53 and 2.54 in simulations 2 and 3 but 2.68 in simulation 1 (*p* < 0.05).

**Table 6:** Root mean squarred error (RMSE) of predictions of HRQoL outcomes for primary ASD and controls over the 10-fold cross validation, with comparison between the 3 simulations.

HRQoL outcomes	Simulation 1 (x-ray)				Simulation 2 (3D movement analysis)				Simulation 3 (x-ray + 3D movement analysis)				Between-simulation comparisons			
	RMSE	SD	95% C.I.		RMSE	SD	95% C.I.		RMSE	SD	95% C.I.		*p*-value	Simulation 1 vs. Simulation 2	Simulation 1 vs. Simulation 3	Simulation 2 vs. Simulation 3
PCS (SF36)	1.68	0.11	1.62	1.75	1.50	0.15	1.41	1.59	1.56	0.16	1.46	1.66	**0.04**	*	*	
MCS (SF36)	1.68	0.05	1.65	1.71	1.50	0.14	1.41	1.59	1.56	0.14	1.47	1.64	**0**.**01**	*	*	
Physical Functioning PF	2.06	0.14	1.98	2.15	1.91	0.12	1.84	1.99	1.92	0.12	1.85	2.00	**0**.**03**	*	*	
Role Physical RP	0.81	0.04	0.79	0.83	0.73	0.07	0.69	0.77	0.74	0.06	0.71	0.78	**0**.**01**	*	*	
Bodily Pain BP	1.72	0.07	1.67	1.76	1.57	0.07	1.53	1.61	1.56	0.08	1.51	1.61	**<0**.**001**	*	*	
General Health GH	1.34	0.09	1.28	1.39	1.20	0.21	1.07	1.33	1.27	0.41	1.01	1.52	0.3			
Vitality VT	2.26	0.14	2.17	2.35	2.08	0.14	1.99	2.17	2.07	0.19	1.95	2.19	**0**.**03**	*	*	
Social Functioning SF	1.91	0.10	1.85	1.97	1.83	0.07	1.78	1.87	1.81	0.08	1.77	1.86	**0**.**04**	** **	*	
Role Emotional RE	0.79	0.04	0.77	0.82	0.72	0.05	0.69	0.74	0.73	0.06	0.69	0.76	**0**.**005**	*	*	
Mental Health MH	2.25	0.07	2.20	2.29	2.01	0.15	1.92	2.11	2.02	0.14	1.93	2.11	**0**.**002**	*	*	
VAS for pain	0.45	0.05	0.42	0.48	0.38	0.03	0.36	0.41	0.32	0.04	0.30	0.34	**0**.**002**	*	*	
ODI	2.68	0.05	2.65	2.71	2.49	0.16	2.39	2.59	2.55	0.14	2.46	2.64	**0**.**03**	*	*	
BDI	1.25	0.05	1.22	1.28	1.06	0.17	0.95	1.16	1.11	0.20	0.99	1.23	**0**.**04**	*		

Bold or *p < 0.05.

**Figure 7:**
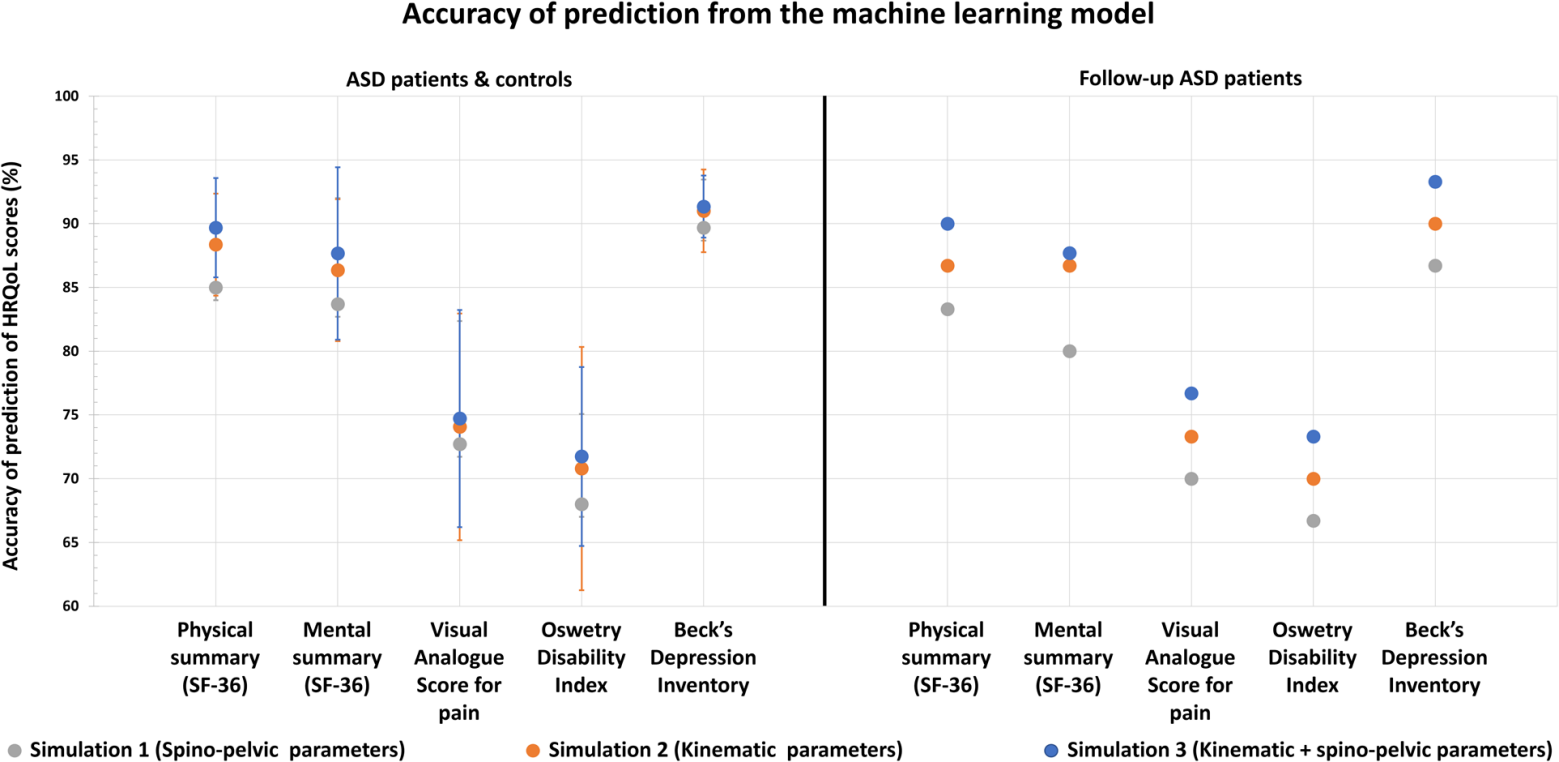
Health related quality of life outcomes and their accuracy of prediction using ML algorithm based on x-ray (spino-pelvic parameters), 3D movement analysis (kinematic parameters) and x-ray + 3D movement analysis combined (spino-pelvic and kinematic parameters).

**Figure 8:**
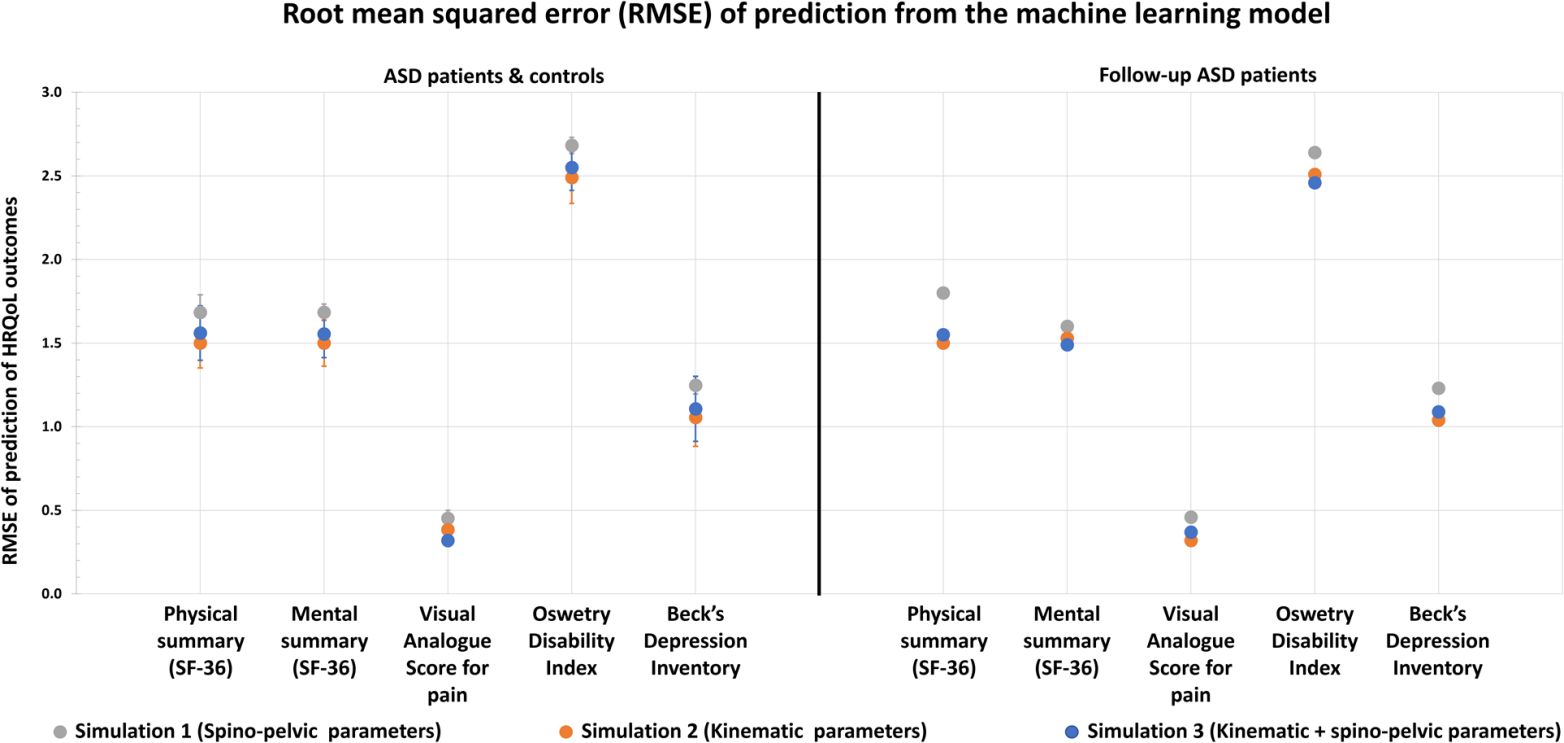
Health related quality of life outcomes and their root mean squared error (RMSE) of prediction using ML algorithm based on x-ray (spino-pelvic parameters), 3D movement analysis (kinematic parameters) and x-ray + 3D movement analysis combined (spino-pelvic and kinematic parameters).

Similar results were reported when the 3 previously mentioned simulations were tested on the follow-up ASD group (Table 7 and Figures 7, 8):

**Table 7:** Accuracy and RMSE of prediction of HRQoL outcomes for the 30 follow-up ASD patients as the testing group.

HRQoL outcomes	Simulation 1 (x-ray)		Simulation 2 (3D movement analysis)		Simulation 3 (x-ray + 3D movement analysis)	
	Accuracy of prediction (%)	RMSE	Accuracy of prediction (%)	RMSE	Accuracy of prediction (%)	RMSE
PCS (SF36)	83.3	1.80	86.7	1.50	90	1.55
MCS (SF36)	80	1.60	86.7	1.53	90	1.49
Physical Functioning PF	80	2.00	80	1.85	83.3	1.85
Role Physical RP	96.7	0.84	93.3	0.71	96.7	0.68
Bodily Pain BP	83.3	1.68	83.3	1.52	86.7	1.55
General Health GH	83.3	1.32	83.3	1.15	86.7	1.18
Vitality VT	70	2.30	73.3	2.02	76.7	2.00
Social Functioning SF	83.3	1.88	86.7	1.79	86.7	1.79
Role Emotional RE	90	0.81	100	0.72	100	0.71
Mental Health MH	76.7	2.27	80	2.00	76.7	2.01
VAS for pain	70	0.46	73.3	0.32	76.7	0.37
ODI	66.7	2.64	70	2.51	73.3	2.46
BDI	86.7	1.23	90	1.04	93.3	1.09

The first simulation with spino-pelvic parameters from the follow-up ASD patients as inputs resulted in a median accuracy of 83.3% (for PCS in SF36), with a maximum of 96.7% (for Role Physical component in SF36) and a minimum of 66.7% (for ODI). A median RMSE of HRQoL outcome prediction of 1.68 was found, with a maximum of 2.64 (for ODI) and a minimum of 0.46 (for VAS for pain).

The second simulation with kinematic parameters from the follow-up ASD patients as inputs resulted in a median accuracy of 85%, with a maximum of 100% (for Role Emotional component in SF36) and a minimum of 70% (for ODI). A median RMSE of HRQoL outcome prediction of 1.5 was found, with a maximum of 2.5 (for ODI) and a minimum of 0.35 (for VAS for pain).

The third simulation combining both kinematic and spinopelvic parameters from the follow-up ASD patients as inputs, resulted in a median accuracy of 86.7% (for MCS in SF36), with a maximum of 100% (for Role Emotional component in SF36) and a minimum of 73.3% (for ODI). A median RMSE of HRQoL outcome prediction of 1.55 was found, with a maximum of 2.48 (for ODI) and a minimum of 0.36 (for VAS for pain).

## Discussion

Spinal deformity is a major cause of quality-of-life deterioration. While classical evaluation of ASD patients is based on full body standing static radiographs and health-related quality of life questionnaires (HRQoL), dynamic evaluation is still missing in their clinical assessment. Previous studies have reported that both radiographic and kinematic alterations correlated with quality of life deterioration (22). More recent studies have shown that gait analysis is a useful tool to objectively quantify patients' function during daily life activities (11, 23). However, it is still unknown if 3D gait analysis is a reliable tool in HRQol scores prediction. This study showed that gait kinematics are great predictors of QoL outcomes and, when combined with radiographic measurements, can explain up to 99% of the variance in QoL presented by these patients.

In an attempt to investigate the main predictors of QoL scores, three machine learning models were computed.

The first simulation revealed that radiographic parameters alone could predict QoL scores, however with suboptimal accuracies (median = 83.4%) and RMSE (median = 1.68). This result is in accordance with previous findings that described postural malalignment as a principal determinant of QoL deterioration in ASD patients (22, 24–26). Other studies reported that surgical restoration of an aligned posture, i.e., restoration of normative or more adapted spino-pelvic and global postural parameters, was associated with improved QoL scores (27).

In the second simulation, gait parameters alone were better predictors of QoL outcomes with a median accuracy of 84.7%, that was significantly higher than the first simulation and a median RMSE of 1.55, that was significantly lower than the first simulation. This is the first study to prove that QoL scores in patients with ASD could be explained and predicted by kinematic parameters collected through gait analysis. The higher accuracies and lower RMSE noted when compared to the first simulation proved that gait kinematics are more representative of function than static radiographic parameters.

In the third simulation, both radiographic and gait parameters were able to successfully predict up to 99% of the alterations of the QoL scores, with a median accuracy of 87%, and a median RMSE of 1.56 showing that patient QoL can be well determined by both static and dynamic parameters simultaneously. This finding also suggests that improving radiographic and gait parameters simultaneously might be a promising way to enhance QoL in these patients.

Nevertheless, the simulation which combined x-ray to 3D gait analysis didn't show neither considerable gain in accuracy of prediction nor considerable loss in RMSE when compared to the simulation based on 3D gait analysis alone. This emphasizes the major role of 3D gait analysis in the assessment of patients with ASD compared to the conventional radiographic assessment.

Furthermore, this study showed that 3D movement analysis is a better predictor than x-Ray of HRQoL outcomes that determine physical consequences of spinal deformity (pain, disability…) estimated by PCS in SF36, VAS for pain and ODI. Moreover, it was interesting to note that 3D movement is also a better predictor of HRQoL outcomes that determine mental repercussions of spinal deformity (depression, anxiety…) on the patient, estimated by MCS in SF36 and BDI. This finding seems to be trivial, since mental health is known to go hand by hand with a patients' ability to perform independently and adequately daily life tasks.

Interestingly, the 3 simulations, performed with the 30 follow-up ASD patients as the testing group, presented similar results. This advocates for the importance of dynamic evaluation, using 3D movement analysis, not only in the primary assessment of patients with ASD but also in the evaluation of surgical and/or medical interventions.

A random forest regressor model was preferred to be used instead of other machine learning models such as SVM or neural networks, since it was more suitable to the aim of this study and the type of data provided, by adopting a systematic decision tree-like approach.

The major limitation of this study relies within not taking into consideration patient's comorbidities which might affect quality of life outcomes and thus being a confounding factor in their prediction.

In conclusion, this study showed that functional assessment, based on 3D movement analysis, is an important predictor of HRQoL outcomes in patients with ASD. Therefore, the assessment of ASD patients should no longer rely on radiographs alone but on movement analysis as well. Knowing that 3D movement analysis is costly, time consuming and requires technological and biomechanical expertise, future research should seek a more practical but valid method to provide kinematic parameters that can be easily used in clinical routine practice.

## Data Availability

The raw data supporting the conclusions of this article will be made available by the authors, without undue reservation.
